# Real-world evaluation of an intravenous iron service for the treatment of iron deficiency with or without anemia

**DOI:** 10.1038/s41598-025-85880-9

**Published:** 2025-04-09

**Authors:** Roel Fijn, Hans C. Ablij, Pieter D. Knoester, Anne M. C. Witte

**Affiliations:** 1 Department of Clinical Pharmacy, Alrijne Healthcare Group, Leiden, The Netherlands; 2 Department of Internal Medicine & Nephrology, Alrijne Healthcare Group, Leiden, The Netherlands; 3 Department of Gastroenterology & Hepatology, Alrijne Healthcare Group, Leiden, The Netherlands; 4 Present Address: Department of Hospital Pharmacy, Northwest Hospital Group, Wilhelminalaan 12, 1815 JD Alkmaar, The Netherlands

**Keywords:** Health care, Medical research

## Abstract

Intravenous (IV) iron is a guideline-recommended treatment for iron deficiency when oral iron is contraindicated, ineffective, or not tolerated, or when rapid iron delivery is necessary. However, evidence suggests that some patients receive less IV iron than needed. This retrospective audit assessed the effectiveness and safety of ferric derisomaltose (FDI), a high-dose IV iron, in 2,468 patients. Efficacy outcomes assessed at 4–12 weeks post-infusion included changes in hemoglobin (Hb) and ferritin, proportion of courses (a course was defined as the treatment episode required to administer one total dose) after which patients were non-anemic (Hb ≥ 130 g/L [men] or ≥ 120 g/L [women]), and response rate (proportion of courses after which patients were non-anemic or Hb increased by ≥ 20 g/L). Safety was assessed through adverse events. Across 2,775 FDI courses, the mean dose was 1,244 mg, but mean estimated iron need was 1,580 mg. At follow-up, mean Hb had increased by 20.9 g/L and mean ferritin by 188.8 µg/L. Patients were non-anemic after 33.4% (n = 494/1,478) of courses and responded after 65.1% (n = 962/1,478) of courses. One patient (n = 1/2,468; 0.04%) had a serious allergic reaction. Patients remained anemic after > 65% of courses, demonstrating the need to optimize dosing based on iron need.

## Introduction

Anemia is a prevalent condition worldwide, affecting ~ 1.8 billion people in 2019^1^. In over half of all cases of anemia, the cause is iron deficiency (ID)^1^. Symptoms of ID and iron deficiency anemia (IDA) include fatigue, headache, dyspnea, and restless leg syndrome^2^, as well as impaired cognitive function, reduced physical performance, and reduced quality of life^3,4^. Both ID and IDA have also been associated with pica (ingestion of non-nutritive substances)^5^. IDA is a frequent complication in patients with conditions in which chronic inflammation or blood loss are present, such as cancer, chronic kidney disease, inflammatory bowel disease, heart failure, and heavy uterine bleeding, as well as being common in pre-operative patients and pregnant women^6–13^. IDA can arise as a result of reduced iron intake, increased iron loss, increased (and unmet) iron demand, and/or impaired iron absorption^14^.

Intravenous (IV) iron is a guideline-recommended treatment for ID/IDA when oral iron preparations are contraindicated, ineffective, or not tolerated, or where there is a clinical need for rapid iron delivery^3,15–22^. Ferric derisomaltose (FDI; Monofer®, Pharmacosmos A/S, Holbæk, Denmark)^23^ is a high-dose IV iron formulation that has demonstrated efficacy and safety in patients with ID/IDA of various etiologies, in numerous clinical trials^24–35^.

In addition to clinical trial data, it is also important to gather real-world evidence to assess the effectiveness of interventions in the clinic^36^, where a broad range of patients are treated and where dosing is determined by local guidance rather than a study protocol. Observational studies assessing real-world data have shown that some patients receive less IV iron than their calculated iron need in clinical practice^37–39^. However, published real-world data in patients with IDA of mixed etiologies are limited. The objective of this study was to evaluate the real-world effectiveness of an established IV iron service provided by the Alrijne Healthcare Group in the Netherlands, by assessing the effectiveness and safety of FDI in patients referred to the service through the use of descriptive statistics. The study also assessed whether real-world dosing with IV iron is in line with product label recommendations. The service evaluation will be used as a tool for improving the implementation and assessment of IV iron therapy across the Alrijne Healthcare Group.

## Methods

### Ethics approval

Local ethics approval for this retrospective study was formally obtained from the board of directors (the local ethics committee) at the Alrijne Healthcare Group (Commissie toetsing lokale haalbaarheid door Raad van Bestuur Alrijne Zorggroep), on the basis that the proposed research did not fall within the scope of the Dutch Medical Research involving Human Subjects Act (Wet medisch-wetenschappelijk onderzoek met mensen; WMO). In this context, due to the nature of the study, the need for informed consent was waived by the local ethics committee. The study was conducted as part of a service evaluation for use in improving the implementation and assessment of IV iron therapy. The study adhered to the principles of the Declaration of Helsinki, the International Council of Harmonisation (ICH), and the ICH Good Clinical Practice Guidelines.

### Study design and patient population

The medical records of all patients who received FDI for the treatment of ID/IDA between January 2014 and December 2021 (inclusive) at the Alrijne Healthcare Group were retrospectively reviewed. Patients were treated if they had a hemoglobin (Hb) level < 130 g/L (men) or < 120 g/L (women), according to the World Health Organization (WHO) definition of anemia^40^, or there was a clinical need to deliver iron rapidly. Ferritin reference values to indicate low iron levels were 25–250 µg/L (men) and 20–250 µg/L (women); however, as normal ferritin levels cannot rule out a deficiency in iron, alongside the Hb cut-offs used, diagnoses of ID/IDA involved a level of clinical judgment. In the Alrijne Healthcare Group, the standard treatment protocol for IV iron includes the simplified dosing method to calculate the iron need of a patient. The simplified dosing method, as per the summary of product characteristics (SPC) for FDI, estimates that, for patients with an Hb level ≥ 10 g/dL (≥ 6.2 mmol/L), total iron need by body weight is: 500 mg (body weight < 50 kg), 1,000 mg (body weight 50– < 70 kg), or 1,500 mg (body weight ≥ 70 kg)^23^. For patients with an Hb level < 10 g/dL (< 6.2 mmol/L), the estimated total iron need, by body weight, is: 500 mg (body weight < 50 kg), 1,500 mg (body weight 50– < 70 kg), or 2,000 mg (body weight ≥ 70 kg)^23^. The total dose of FDI per week should not exceed 20 mg iron/kg body weight, and a single infusion should not exceed 20 mg iron/kg body weight^23^. In acute situations where patient Hb data are not available, but where there is a rapid need for iron, the standard treatment protocol recommends that a fixed dose of 1,000 mg of FDI be administered. The protocol for IV iron therapy for patients with gynecological complications or for those undergoing hemodialysis differs from the standard treatment protocol used at the Alrijne Healthcare Group. Therefore, the records of patients who had been referred from gynecology departments or were undergoing hemodialysis were excluded from this audit. Patients with no follow-up data due to death were also excluded.

As part of routine testing, Hb and ferritin levels were measured at baseline (i.e., before FDI administration) and re-assessed at follow-up, which was 4–12 weeks post-infusion (i.e., post-baseline). Patients without ferritin data pre- and post-infusion were excluded from this study.

### Data collection and outcomes

Data for patient demographics, iron dose, Hb and ferritin levels, and adverse events (AEs) were collected from patient medical records and analyzed. Follow-up data outside the period 4–12 weeks post-infusion were not collected. The dose of FDI was evaluated by comparing the actual iron dose administered with the estimated total iron need. Estimated total iron need was calculated using the simplified dosing method, based on individual patient baseline Hb and weight and according to the SPC for FDI^23^. A course of IV iron was defined as the treatment episode required to administer a total dose; the total dose was administered either in a single infusion or split over two infusions that were administered one week apart.

The main efficacy outcome was the change in Hb from baseline to 4–12 weeks post-infusion. Additional efficacy outcomes were the change in ferritin from baseline to 4–12 weeks post-infusion; the proportion of courses after which patients were non-anemic (i.e., had an Hb level ≥ 130 g/L [men] or ≥ 120 g/L [women], in alignment with the WHO definition of anemia)^40^ at 4–12 weeks post-infusion; and response rate (defined as the proportion of courses after which patients were non-anemic as per WHO definition^40^ or after which patients experienced an Hb increase of ≥ 20 g/L from baseline) at 4–12 weeks post-infusion. Anemia status was evaluated in patients receiving their estimated total iron need, and in those who received less or more than their estimated total iron need. Safety was assessed through AEs.

### Data analysis

All data were analyzed descriptively using Microsoft Excel (Microsoft Corp., Washington, US).

Patients with no Hb and weight data pre-infusion, or missing dose information, were excluded from analyses involving Hb assessment.

## Results

### Patient population

Baseline demographics and clinical characteristics were collected for 2,468 patients (Table 1).

**Table 1:** Baseline demographics and clinical characteristics.

Demographics	All patients (N = 2,468)
Gender, n (%)	
Female	1,524 (61.8)
Male	944 (38.2)
Age at which the first course of IV iron was administered, years	
Mean (SD)	68 (18.4)
Range	16–103
Weight, kg	n = 2,265
Mean (SD)	76.7 (18.2)
Range	35.9–196
Referral, n (%)	
Internal Medicine^a^	1,596 (64.7)
Gastroenterology–Hepatology	476 (19.3)
Geriatrics	135 (5.5)
Cardiology	96 (3.9)
Pulmonary	52 (2.1)
Other	113 (4.6)

Clinical characteristics	All IV iron courses (N = 3,481)
Hb, g/L	n = 2,954
Mean (SD)	96.1 (18.5)
Range	40.25–162.61
Anemia status,^b^ n (%)	n = 2,954
Anemic	2,786 (94.3)
Non-anemic	168 (5.7)
Ferritin, µg/L	n = 1,622
Mean (SD)	36.2 (88.0)
Range	1–1,505
Serum iron, mmol/L	n = 1,276
Mean (SD)	6.9 (15.0)
Range	1–266
Previous IV iron exposure within the audit period, n (%)	n = 2,454
Yes	450 (18.3)
No	2,004 (81.7)

Some patients received multiple courses of IV iron so baseline clinical data prior to each course are included.

^a^Included referrals from Nephrology, Endocrinology, Vascular Medicine, Infectiology, and Hematology–Oncology departments; ^b^anemia was defined as Hb < 130 g/L (men) or < 120 g/L (women). *Hb* hemoglobin, *IV* intravenous, *SD* standard deviation.

The study population was predominantly female and represented patients referred from internal medicine, gastroenterology–hepatology, geriatrics, cardiology, pulmonary, and other departments. Before the majority of IV iron courses, patients were anemic as per the WHO definition^40^; the majority of patients had not previously received IV iron during the audit period.

### Intravenous iron dosing

A total of 3,481 courses of FDI were administered to 2,468 patients during the audit period. Follow-up Hb data at 4–12 weeks post-infusion were available for 1,478 courses. Across all courses where patients had Hb and weight data pre-infusion (n = 2,775 courses), the mean actual dose of FDI administered was 1,244 mg. Based on pre-infusion baseline Hb and weight, the mean estimated total iron need was calculated to be 1,580 mg, demonstrating that iron dosing was not always consistent with the simplified dosing method that is included in the standard treatment protocol. Overall, 1,654 (59.6%) courses provided patients with less than their estimated total iron need, 741 (26.7%) courses provided patients with their estimated total iron need, and 371 (13.4%) courses provided patients with more than their estimated total iron need. It was not possible to determine the adequacy of iron dosing for 9 (0.3%) courses for various reasons, including premature termination of administration and unclear dosing information in the records. The level of underdosing was similar throughout the study duration and across specialties.

### Efficacy

Following treatment with FDI, the mean time to Hb follow-up was 44 days. For the 1,478 courses where follow-up Hb data were available, mean Hb levels had increased by 20.9 g/L (to 117.0 g/L) at follow-up. For the 681 courses where follow-up ferritin data were available, mean ferritin levels had increased by 188.8 µg/L (to 225.0 µg/L) at follow-up.

When stratified according to actual dose of iron administered, mean Hb levels increased across all groups: by 23.8 g/L following courses (n = 925 at follow-up) that provided patients with less than their estimated total iron need, by 21.6 g/L following courses (n = 385 at follow-up) that provided patients with their estimated total iron need, and by 18.7 g/L following courses (n = 168 at follow-up) that provided patients with more than their estimated total iron need. Mean time to follow-up Hb assessment was 41 days for patients who received less than their total iron need, 44 days for patients who received their total iron need, and 48 days for patients who received more than their total iron need.

Mean ferritin levels increased across all groups stratified according to actual dose of iron administered: by 142.0 µg/L following courses (n = 389 at follow-up) that provided patients with less than their estimated total iron need, by 206.8 µg/L following courses (n = 201 at follow-up) that provided patients with their estimated total iron need, and by 326.8 µg/L following courses (n = 91 at follow-up) that provided patients with more than their estimated total iron need.

**Fig. 1:**
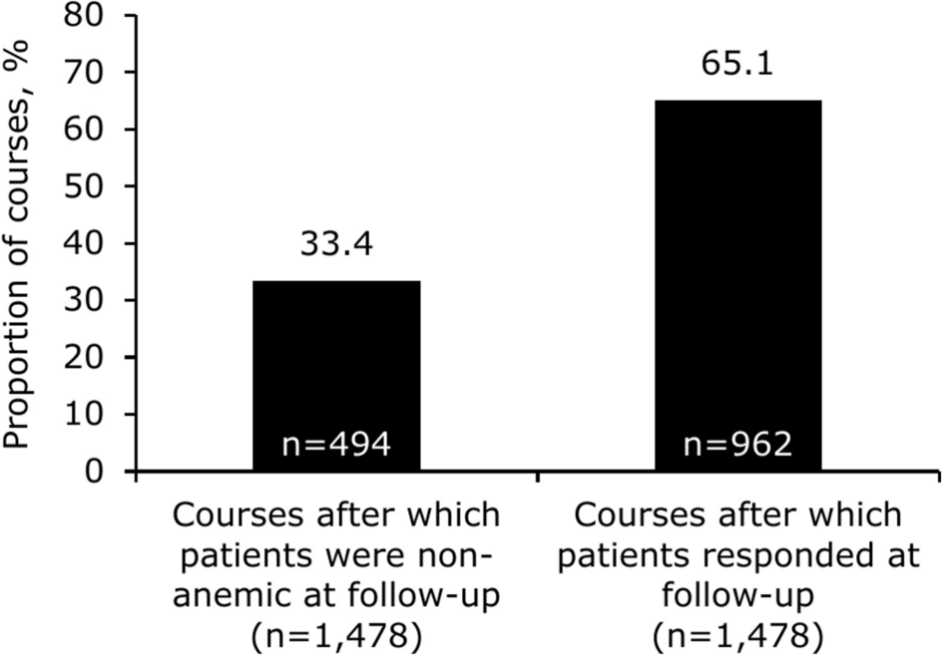
Proportion of FDI courses after which patients were non-anemic or responded at follow-up. Patients were non-anemic at baseline for 5.7% of courses (n = 168/2,954). Non-anemic status was defined as Hb ≥ 130 g/L (men) and ≥ 120 g/L (women). Response rate was defined as the proportion of courses after which patients were non-anemic as per WHO definition ^40^ or after which patients experienced an Hb increase of ≥ 20 g/L from baseline. Mean time to follow-up was 44 days. *FDI* ferric derisomaltose, *Hb* hemoglobin, *WHO* World Health Organization.

The proportion of courses after which patients were non-anemic or responded at follow-up is shown in Fig. 1.

**Fig. 2:**
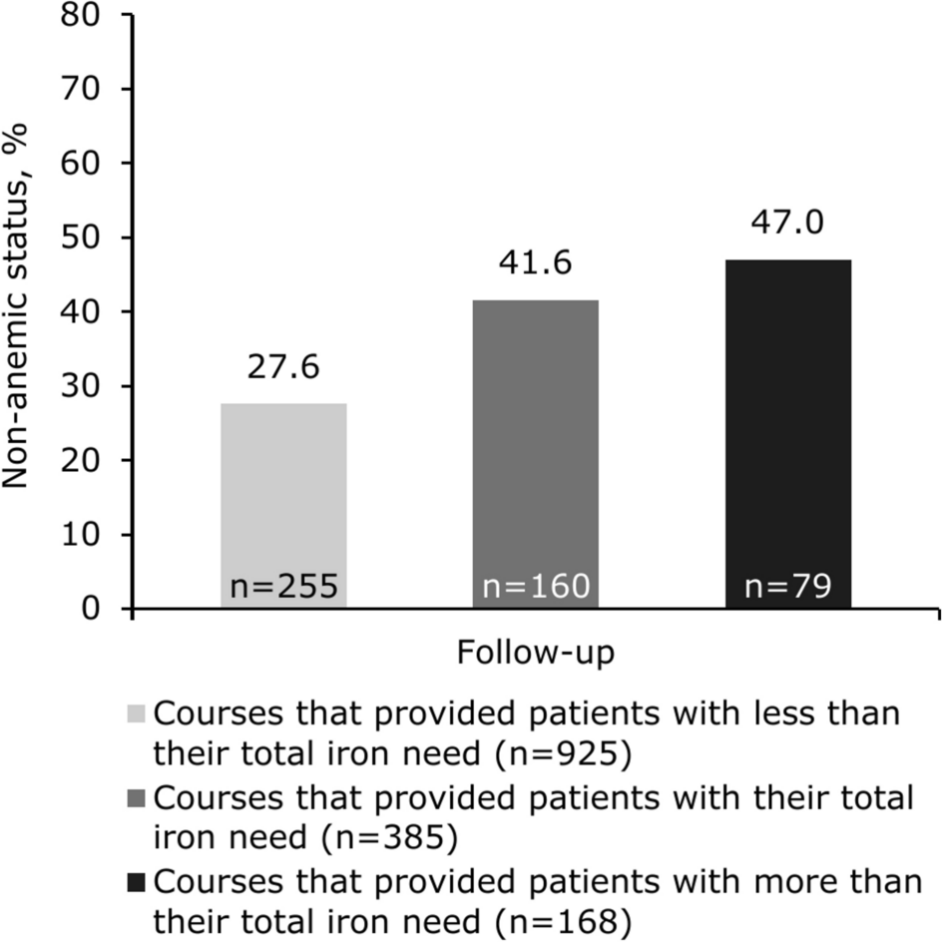
Proportion of FDI courses after which patients were non-anemic at follow-up, according to actual dose of iron administered. Non-anemic status was defined as Hb ≥ 130 g/L (men) and ≥ 120 g/L (women). Mean time to follow-up was 41 days for patients who received less than their total iron need, 44 days for patients who received their total iron need, and 48 days for patients who received more than their total iron need. *FDI* ferric derisomaltose, *Hb* hemoglobin.

The proportion of courses after which patients were non-anemic at follow-up, according to actual dose of iron administered, is shown in Fig. 2.

### Safety

AEs occurred in 3.1% of patients (n=76/2,468) and are listed in Table 2.

**Table 2:** Adverse events.

	All patients (N = 2,468)
**All AEs, n (%) [E]**	n = 76 (3.1) [97]
**AEs by system organ class, n (%)** ^**a**^	
Immune system disorders	
Hypersensitivity	12 (0.49)
Serious allergic reaction^b^	1 (0.04)
Nervous system disorders	
Headache	3 (0.12)
Loss of consciousness	1 (0.04)
Dizziness	6 (0.24)
Cardiac disorders	
Tachycardia	1 (0.04)
Vascular disorders	
Hypotension	2 (0.08)
Respiratory, thoracic, and mediastinal disorders	
Chest pain	2 (0.08)
Chest tightness	14 (0.57)
Dyspnea	8 (0.32)
Wheezing	2 (0.08)
Gastrointestinal disorders	
Nausea	10 (0.41)
Abdominal pain	2 (0.08)
Vomiting	1 (0.04)
Diarrhea	1 (0.04)
Skin and subcutaneous tissue disorders	
Rash	4 (0.16)
Urticaria	2 (0.08)
Flushing	9 (0.36)
Sweating	2 (0.08)
Distant skin discoloration	2 (0.08)
Musculoskeletal and connective tissue disorders	
Muscle spasms	2 (0.08)
General disorders and administration site conditions	
Injection site reactions^c^	2 (0.08)
Malaise	1 (0.04)
Flu-like symptoms	3 (0.12)
Other	
Bone pain	1 (0.04)
Leg pain	1 (0.04)
Burning throat	1 (0.04)
Unspecified AE	1 (0.04)

^a^Each AE was recorded once per patient; ^b^an AE was classified as a serious allergic reaction if treatment with epinephrine was required; ^c^includes the following preferred terms: injection site erythema, injection site swelling, injection site burning, injection site pain, injection site bruising, injection site discoloration, injection site extravasation, injection site irritation, injection site reaction. *AE* adverse event, *E* number of adverse events, *n* number of patients experiencing an adverse event.

Less than 1% of patients (n = 12/2,468) experienced a hypersensitivity reaction (HSR); these reactions were treated with steroids and/or antihistamines. One patient experienced a serious allergic reaction, which was additionally treated with epinephrine. It was not possible to verify whether the serious allergic reaction was indeed an anaphylactic reaction.

## Discussion

This retrospective study provides real-world data for the efficacy and safety of FDI in patients referred to an established IV iron service from various hospital departments within the Alrijne Healthcare Group in the Netherlands. Overall, where follow-up data were available, increases in Hb and ferritin levels were observed in patients treated with FDI during the audit period, regardless of the dose of iron received by patients relative to their estimated total iron need. The safety profile of FDI was favorable, with a very low incidence of AEs, including HSRs. These findings support those of numerous randomized controlled trials (RCTs), which have shown that FDI is efficacious and well-tolerated in patients with ID/IDA of various etiologies^24–34^.

The results of this retrospective study also align with existing real-world evidence of the efficacy and safety of FDI in clinical practice^39,41,42^. In this study, mean Hb levels had risen substantially at 4–12 weeks post-infusion. However, after more than 65% of courses, patients remained anemic, especially those that were underdosed. This likely indicates a higher iron need, though other causes of anemia (such as chronic gastrointestinal bleeding) cannot be ruled out, due to the retrospective nature of this study. Additionally, ferritin levels rose the least in patients that received less IV iron than their estimated total iron need. A previous service evaluation that investigated the real-world use of FDI for the treatment of ID/IDA in patients with gastroenterological disorders reported a drop in ferritin at the second follow-up visit (median 181 days) after initially observing an increase in ferritin at the first follow-up visit (median 30 days)^39^. As postulated by Kearns & Jacob (2021), and in light of the observation that an increase in Hb was sustained, the decrease in ferritin may have been the result of iron being used up in hematopoiesis^39^. Similarly, in this study, the low rise in ferritin levels in patients who received less IV iron than their estimated total iron need may suggest that iron is being utilized to replenish Hb levels, and that iron stores are not being replenished sufficiently. Furthermore, patients who receive less iron than their estimated iron requirement may be at an increased likelihood of requiring an additional course of IV iron. Prospective, real-world, observational studies have shown that patients who received a higher initial dose of FDI (> 1,000 mg) had a substantially reduced probability (54–65%) of requiring re-dosing with FDI compared with patients whose first dose was 1,000 mg or less^37,41^. Notably, almost 60% of courses provided patients with less than their estimated total iron need in this study; existing real-world evidence has also highlighted that patients are frequently underdosed^37,38,41^. Given these observations, consistent use of the simplified dosing method is important to ensure patients receive their estimated iron need, and consideration of repeat IV iron dosing and additional follow-up assessments may be beneficial. Further to this, the results show that the mean actual dose of FDI administered was lower than the mean estimated total iron need that was calculated based on pre-infusion baseline Hb and weight (1,244 mg versus 1,580 mg). Generally, in most patients, the calculated iron need can be supplied in one infusion, as the total dose of FDI in a single infusion can be up to 20 mg iron/kg body weight^23^. Accordingly, opportunities exist for providing patients with their estimated total iron need in one hospital visit, with no requirement for extra resource or hospital capacity utilization.

In this study, the incidence of AEs was low for all patients (3.1%), with HSRs reported in 12 patients (< 0.5%) and a serious allergic reaction in one patient only (< 0.05%). Observational studies conducted across sites in the UK, Denmark, Norway, and Sweden have reported similarly low rates of adverse drug reactions (0.3–4%) and no or very few serious allergic reactions (0–0.1%) following FDI treatment^37–39,41,42^. In contrast to the very low incidence of HSRs reported in this study, a single-center cohort study that used prospectively developed registration forms to record HSRs has reported a higher incidence of HSRs after FDI administration, at 8.7%^43^. However, the study may have been affected by information bias, as healthcare personnel had experience with another IV iron product before switching to FDI use, which may have increased awareness of HSRs when introducing a different IV iron^43^.

The safety data in the present study add to the evidence that moderate-to-severe or serious HSRs with modern IV iron preparations are extremely rare^44–46^. In a pooled analysis of five RCTs that investigated the incidence of moderate-to-severe or serious HSRs after treatment with any of the four most commonly used IV iron formulations in Europe and the US (ferric carboxymaltose [FCM], FDI, ferumoxytol, iron sucrose), the overall incidence of moderate-to-severe or serious HSRs was 0.2–1.7%^44^. In a meta-analysis that included data from 103 RCTs, no IV iron preparation (aside from ferric gluconate) was associated with a significantly increased risk of severe infusion reactions relative to comparators (i.e., placebo, no iron, oral iron, or intramuscular iron)^47^. A more recent meta-analysis of 15 RCTs reported a lower incidence of HSRs with FDI versus FCM (0.14% versus 1.08%), and found that the odds of experiencing a serious or severe HSR were significantly reduced with FDI compared with FCM^46^.

The majority of AEs following IV iron are minor and self-limited infusion reactions (e.g., Fishbane reactions)^48^. However, it has been noted that, with limited clinical experience, minor reactions can be misinterpreted as impending anaphylaxis, leading to unnecessary intervention with epinephrine or antihistamines, which can, in turn, exacerbate the otherwise mild and self-limited reaction^48^. Therefore, it is challenging to identify anaphylactic reactions consistently across clinics. Given that the term ‘anaphylaxis’ is not always used appropriately^48^, we have instead used the term ‘serious allergic reaction’ to describe the single HSR that was treated with epinephrine. As most reactions to IV iron are mild and self-limiting in nature, and many patients can be successfully re-challenged with IV iron, it is important to recognize the symptoms of different types of immediate infusion reactions and implement an appropriate management strategy^44^. An algorithm has been proposed to assist healthcare professionals in the management of acute infusion reactions, including serious allergic reactions^44^.

Hypophosphatemia and its clinical manifestations (e.g., fatigue, muscle weakness, osteomalacia) are a known potential side effect of certain IV iron products^49^. Phosphate levels were not routinely measured in the presented study population. However, no cases of hypophosphatemia in relation to FDI treatment were reported in the present study. This is consistent with previous findings of a low risk of hypophosphatemia with FDI^33,34^.

This study has several limitations, some of which are inherent to its retrospective design. First, data on the cause of ID/IDA and full details on how ID/IDA was diagnosed were not available. Second, unlike Hb and ferritin, transferrin saturation was not measured as part of routine testing. Follow-up Hb and ferritin data were not available after a large proportion of courses, possibly because many patients were followed up in a primary care setting. Third, the study included only one follow-up assessment (4–12 weeks post-infusion; mean time to follow-up: 44 days), so it is difficult to assess when the peak Hb response occurred. Fourth, despite use of a local treatment protocol for the administration of FDI, iron dosing was not always consistent with the simplified dosing method, which may have contributed to the difference between the number of courses that provided patients with IV iron less than, equal to, or more than their estimated total iron need. Despite these limitations, the strengths of the study lie in evaluating the effectiveness and safety of FDI for the treatment of ID/IDA in a large mixed patient population, using real-world data.

In conclusion, this study adds to the existing real-world evidence of the efficacy and safety of FDI treatment across diverse patient populations treated in the clinic. Despite increases in mean Hb levels in this study, after more than 65% of IV iron courses, patients remained anemic, particularly in the group that received less IV iron than their estimated total iron need. Therefore, consistent use of the simplified dosing method is important to ensure patients receive their estimated iron need, and consideration of repeat IV iron dosing and additional follow-up assessments may be called for. Consequently, as a method to ensure on-label prescription of IV iron in terms of the correct doses in relation to body weight and Hb levels, standardized digital prescriptions of IV iron have been introduced into the hospital group’s computerized prescription order entry system.

## Data Availability

All processed data are available upon reasonable request from the corresponding author.
